# Pharmacokinetic Properties of Acetyl Tributyl Citrate, a Pharmaceutical Excipient

**DOI:** 10.3390/pharmaceutics10040177

**Published:** 2018-10-08

**Authors:** Hyeon Kim, Min Sun Choi, Young Seok Ji, In Sook Kim, Gi Beom Kim, In Yong Bae, Myung Chan Gye, Hye Hyun Yoo

**Affiliations:** 1 Institute of Pharmaceutical Science and Technology and College of Pharmacy, Hanyang University, Ansan, Gyeonggi-do 15588, Korea; kimhyeon2000@nate.com (H.K.); chm2456@hanyang.ac.kr (M.S.C.); wldudtjr23@hanyang.ac.kr (Y.S.J.); kis@hanyang.ac.kr (I.S.K.); shbn13@naver.com (G.B.K.); iybae722@naver.com (I.Y.B.); 2 Department of Life Science, Institute of Natural Sciences, Hanyang University, Seoul 04763, Korea

**Keywords:** acetyl tributyl citrate, pharmaceutical excipient, pharmacokinetics, metabolic stability, plasma

## Abstract

Acetyl tributyl citrate (ATBC) is an (the Food and Drug Administration) FDA-approved substance for use as a pharmaceutical excipient. It is used in pharmaceutical coating of solid oral dosage forms such as coated tablets or capsules. However, the information of ATBC on its pharmacokinetics is limited. The aim of this study is to investigate the pharmacokinetic properties of ATBC using liquid chromatography–tandem mass spectrometric (LC–MS/MS) analysis. ATBC was rapidly absorbed and eliminated and the bioavailability was 27.4% in rats. The results of metabolic stability tests revealed that metabolic clearance may have accounted for a considerable portion of the total clearance of ATBC. These pharmacokinetic data would be useful in studies investigating the safety and toxicity of ATBC.

## 1. Introduction

Plasticizers are chemicals which are added into polymeric materials to increase their flexibility and workability. They are also used in polymers for pharmaceutical applications such as drug carriers and coatings. In the chemical industry, many kinds of plasticizers are utilized. However, for pharmaceutical application, the use of only several kinds of plasticizers has been approved as it requires low toxicity and migration. Pharmaceutically used plasticizers can be categorized into hydrophobic and hydrophilic groups [1]. The hydrophobic plasticizers include acetyl tributyl citrate (ATBC), acetyl triethyl citrate, castor oil, diacetylated monoglycerides, dibutyl sebacate, diethyl phthalate, triacetin, tributyl citrate, and triethyl citrate. The hydrophilic plasticizers include clycerin, polyethylene glycols, polyethylene glycols monomethyl ether, propylene glycol, and sorbitol sorbitan solution.

Among the plasticizers mentioned above, ATBC is a biodegradable plasticizer formed by the esterification of citric acid. ATBC is used in pharmaceutical coating of solid oral dosage forms such as coated tablets or capsules. According to the online (the Food and Drug Administration) FDA database, some oral drugs contain 0.56~57.55 mg of ATBC [2]. For example, Cardizem CD (a coated capsule for extended release containing diltiazem hydrochloride) contains ATBC; if the maximum dose of Cardizem CD (480 mg) is given to patients, they may be exposed to approximately 20 mg of ATBC per day [2]. 

Generally, citric acid trimesters are known to be very safe. The toxicity of ATBC has been investigated thoroughly in animals. Research showed that the acute oral LD50 for ATBC in mice or rats was greater than 25 g/kg and that ATBC was not associated with any significant reproductive toxicity in mice or rats [3]. The European Commission stated that the no-observed adverse effect level of ATBC was 100 mg/kg bw/day (C7/GF/csteeop/ATBC/080104 D(04)). The European Food Safety Authority has given ATBC a tolerable daily intake value of 1.0 mg/kg bodyweight [4]. Based on these reports, ATBC is considered toxic only at very high concentrations. However, one study reported that ATBC induced intestinal Cytochrome P450 3A4 (CYP3A4) at a relatively low concentration (about 4.02 µg/mL) [2]. In addition, studies published recently suggested that ATBC may affect female reproduction at a low dose [5]. Therefore, human exposure to ATBC levels needs to be monitored, and a better understanding of the pharmacokinetics of ATBC is needed.

There is a report on the safety of ATBC which evaluated the absorption, metabolism, and excretion profiles of ATBC using 14C-labeled compounds [2]. This report provides the overall pharmacokinetic properties of ATBC but the detailed pharmacokinetic information on ATBC, an unchanged form, is unavailable.

Many studies have described analytical methods for the determination of ATBC in plastic materials. These analytical methods include gas chromatography–mass spectrometry (GC–MS), GC with a flame ionization detector, high-performance liquid chromatography (HPLC) with an ultraviolet/visible spectroscopic detector, HPLC with an evaporative light scattering detector, and nuclear magnetic resonance spectroscopy [6,7,8,9]. Recently, a liquid chromatography–tandem mass spectrometric (LC–MS/MS) method has been reported [10]. However, there are no analytical methods for the determination of ATBC in biological samples. 

In the present study, we developed an analytical method for the pharmacokinetic analysis of ATBC using liquid chromatography–tandem mass spectrometry (LC–MS/MS). Based on the developed method, we investigated the pharmacokinetic properties of ATBC in vitro and in vivo.

## 2. Materials and Methods

### 2.1. Chemicals and Materials

ATBC (>98%), acetyl triethyl citrate (ATEC, internal standard) and PMSF (esterase inhibitor) were purchased from Sigma-Aldrich (St. Louis, MO, USA). HPLC grade water was generated using a Milli-Q purification system (Millipore, Bedford, MA, USA). HPLC grade methanol (MeOH) and acetonitrile (ACN) was purchased from J.T. Baker (Phillipsburg, NJ, USA). Blank plasma was collected in a heparin tube from male Sprague-Dawley rats. All chemicals and solvents were of analytical grade.

### 2.2. Preparation of Calibration and Quality Control Standards

A stock solution (5 mg/mL) of ATBC was prepared in 70% acetonitrile. The stock solution was stored at −20 °C and brought to room temperature before use. Working standard solutions were prepared to final concentrations ranging from 0.1 µg/mL to 10 µg/mL with acetonitrile. An internal standard (IS, ATEC) solutions (5 mg/mL) was prepared at 5 mg/mL and further diluted to a concentration of 100 ng/mL in methanol. Calibration samples (10, 50, 100, 200, 500 and 1000 ng/mL) were prepared by spiking 90 µL of rat plasma with the appropriate working standard solution of the 10 µL. Four levels of quality control (QC) samples were prepared at 10, 30, 400 and 800 ng/mL in the same way as the calibration standards. All the prepared plasma samples were stored at −20 °C until use. Calibration standards and QC samples were added with 3 volumes of the IS solution for protein precipitation, vortex-mixed for 30 s, and centrifuged at 12000 rpm for 5 min at 4 °C. The supernatant was collected and transferred to an LC vial for LC-MS/MS analysis.

### 2.3. Analytical Method Validation

The method was validated according to the US Food and Drug Administration Bioanalytical Method Validation Guidance [11,12]. The selectivity was evaluated by comparing the chromatograms of six individual blank plasmas with corresponding standard-spiked plasma samples. Calibration curves were constructed by plotting the peak area ratios of the analyte to the IS versus the analyte concentrations. The equation model was obtained by weighted least squares linear regression analysis. The lower limit of quantification (LLOQ) was determined as the lowest concentration with a relative standard deviation (RSD) of <20% and with 80–120% accuracy. The intra- and inter-day accuracy and precision were evaluated by analyzing replicates (*n* = 5 for intra-day; *n* = 3 for inter-day) of spiked plasma samples at concentrations of 10, 30, 400 and 800 ng/mL. The extraction recovery was defined by comparing the peak area of the pre-spiked extracted sample with that of the post-spiked extracted sample. The effect of plasma matrix was evaluated by comparing the peak area of the post-spiked extracted sample with that of the neat solution. Six different lots of blank plasma were evaluated. The stability of the analyte was determined: Freeze and thaw, bench-top, long-term, and post-preparative stability. 

### 2.4. Pharmacokinetics Study in Rats

The developed and validated method was applied to a pharmacokinetic study of ATBC in rats after oral administration. Male Sprague-Dawley rats (8 weeks, 257 ± 20 g) were purchased from Orient Bio (Seongnam, Korea) and maintained at a temperature of 23 ± 3 °C with a 12 h light-dark cycle and moisture (55 ± 15% relative humidity) controlled room. Before dosing, the rats had fasted for 12 h and had free access to water. The rats were administered ATBC in 30% PEG intravenously (10 mg/kg) and orally (500 mg/kg). Blood samples (200 μL) were collected from the carotid artery into tubes containing heparin: Esterase inhibitor (1 M PMSF) was used to prevent degradation in rat plasma. Blood samples were collected at 1, 2, 5, 10, 15, and 30 min and 1, 2, 4, 6, 10, and 24 h for IV injection and 5, 15, 30, and 45 min and 1, 2, 4, 6, 8, 10, and 24 h for oral administration. Plasma samples, obtained by centrifuging blood at 12,000 rpm for 5 min at 4 °C. The supernatant plasma (50 μL) was collected in a separate microtube and treated with 150 μL methanol with IS (100 ng/mL of ATEC). The samples were vortexed and centrifuged at 12,000 rpm for 5 min. The supernatant was analyzed using LC-MS/MS. All animal procedures were approved by the Institutional Animal Care and Use Committee of Hanyang University (2016-0235A).

### 2.5. Pharmacokinetics Analysis

The pharmacokinetic parameters of ATBC were calculated by using the noncompartmental analysis of the Phoenix WinNonlin Enterprise Program v5.3 (Pharsight Inc., St. Louis, MO, USA). Data were shown as mean ± standard deviation. The following pharmacokinetic parameter were calculated. Maximum plasma concentration (Cmax) and the time to reach maximum (Tmax) were determined directly from the measured data. The elimination half-life (*t*_1/2_) was calculated as 0.693/λz. The terminal elimination rate constant (λz) was determined by linear least-squares regression of the terminal portion of the logged plasma concentration-time curve. The area under the plasma concentration-time curve (AUC) from time 0 to the time of the last measurable concentration (AUC0 → t) was calculated using the log-linear trapezoidal rule. In the IV dosing data, the distribution volumes (Vd) based on the terminal phase and the total-body clearance (Cl) were obtained directly from the experimental data.

### 2.6. Metabolic Stability Assay

ATBC was incubated at 37 °C for 0, 30, 60, and 120 min in rat and human plasma and liver microsomes. The detailed procedures are described in previously published reports [13]. In addition, to characterize the enzymes which may be involved in microsomal metabolism of ATBC, ATBC was incubated at 37 °C for 60 min with cDNA expressed human CYP isoforms. The final concentration of each CYP isozyme was 0.05 nmol/mL. After incubation, the sample was treated as described in 2.4. All experiments were performed in triplicate. The concentration of ATBC was measured using LC-MS/MS analysis. 

### 2.7. LC-MS/MS Conditions

**Figure 1:**
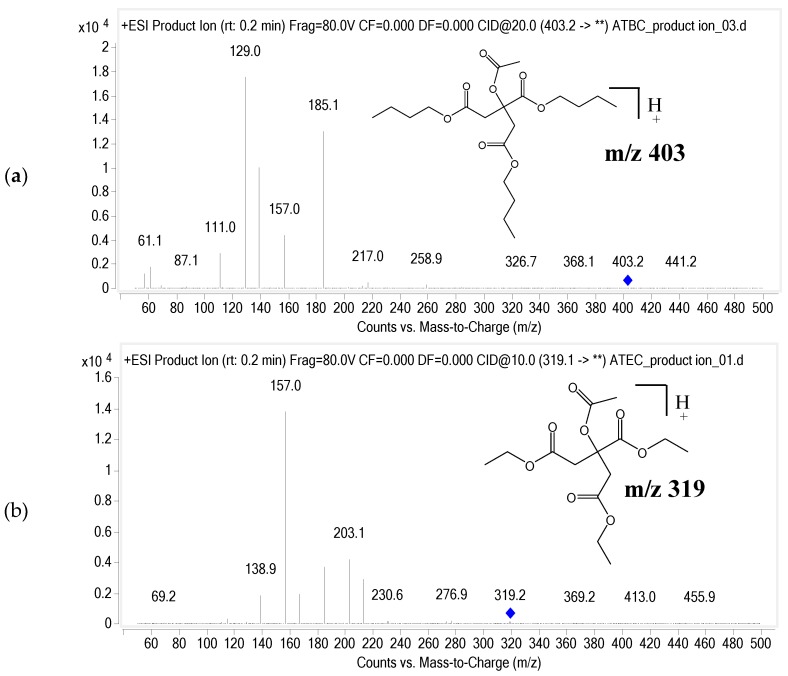
Product ion mass spectrum of (**a**) Acetyl tributyl citrate (ATBC) and (**b**) Internal standard (IS) (ATEC, acetyl triethyl citrate).

The LC/MS/MS system consisted of a 6460 triple quadrupole mass spectrometer (Agilent Technologies, Santa Clara, CA, USA) coupled with the Agilent 1260 infinity HPLC system. The column temperature was maintained at 30 °C using a thermostatically controlled column oven. The mobile phases consisted of 0.1% formic acid in DW (solvent A) and 0.1% formic acid in 90% ACN (solvent B). A gradient elution was performed at a flow rate of 0.3 mL/min and initiated with 10% of mobile phase B, increased to 100% for 1 min and held for 3 min and restored to the initial percentage for 2 min to re-equilibrate. Chromatographic separation was achieved with a Kinetex C18 (2.1 mm × 50 mm, 2.6 μm; Phenomenex). The electrospray ionization (ESI) source was operated in positive mode. Multiple reaction monitoring (MRM) was employed. The precursor-product ion pairs used were 403.2 *m*/*z* → 129.0 *m*/*z* for ATBC and 319.1 *m*/*z* → 157.0 *m*/*z* for ATEC. The fragmentor voltages were 80 V and 70 V, and the collision energy were 20 V and 10 V for ATBC and ATEC, respectively. The product ion spectrum of ATBC and ATEC is presented with its chemical structure in Figure 1a,b.

## 3. Results

### 3.1. Analytical Method Validation

ATBC has three ester bonds in its structure. Generally, compounds with esters are susceptible to hydrolysis in plasma, making it difficult to analyze ATBC in plasma samples [14,15]. Accordingly, pretreatment is required to ensure the stability of ATBC in plasma samples. We utilized phenylmethylsulfonyl fluoride (PMSF) as an esterase inhibitor in the pretreatment step, which enabled an accurate and reproducible analysis of ATBC in plasma.

#### 3.1.1. Selectivity

**Figure 2:**
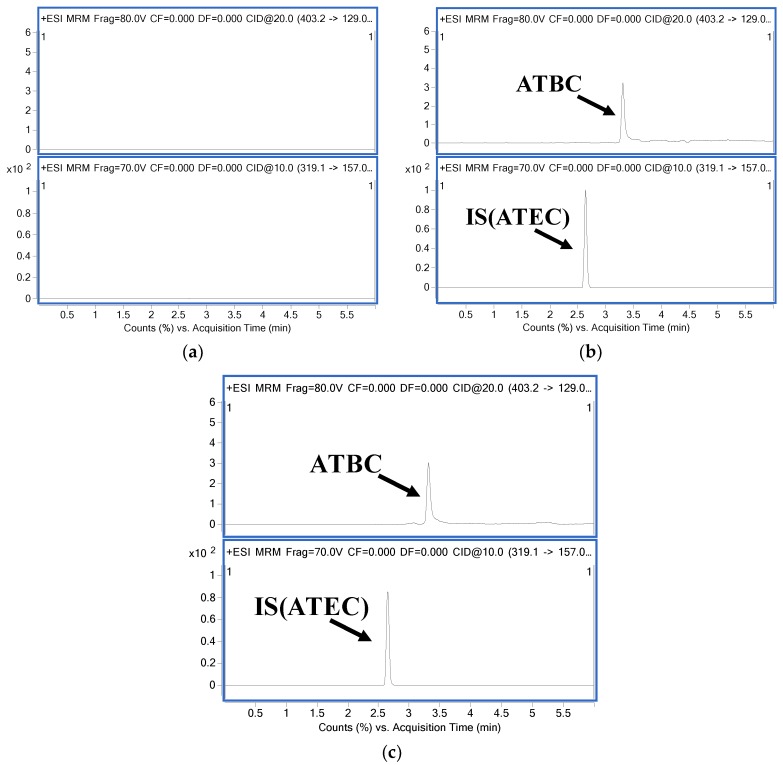
Representative multiple reaction monitoring (MRM) chromatograms of ATBC and IS (ATEC) in rat plasma. (**a**) blank plasma; (**b**) The lower limit of quantification (LLOQ) of ATBC at 10 ng/mL and IS at 100 ng/mL; (**c**) plasma sample at 10 h after oral (PO) administration of 500 mg/kg of ATBC.

Typical chromatograms of blank plasma, blank plasma spiked with ATBC (10 ng/mL) and ATEC (IS; 100 ng/mL), and rat plasma samples 15 min after PO administration of ATBC are shown in Figure 2. The retention times of ATBC and ATEC (IS) were 3.5 min and 2.7 min, respectively. As shown in Figure 2, endogenous plasma components did not interfere with the retention times of the analyte and IS.

#### 3.1.2. Linearity

Calibration curves using six concentration levels (10, 50, 100, 200, 500 and 1000 ng/mL) with eight replicates each were constructed to evaluate the calibration model. The regression coefficient (r2) was ≥0.990. The accuracy and precision of the calibration standard curves were reliable (less than ±15% RSD) for all the concentration points tested.

#### 3.1.3. Accuracy and Precision

The results for intra-day and inter-day precision and accuracy in plasma QC samples at concentrations of 10, 30, 400, and 800 ng/mL are summarized in Table 1. The intra- and inter-day precision was within 11.4%. The intra-day accuracy was 90.7–117.6%, whereas the inter-day accuracy was 100.2–103.4%. 

#### 3.1.4. Extraction Recovery and Matrix Effect

The mean extraction recovery of ATBC from rat plasma was 85.5 ± 9.0% and 93.8 ± 12.0% at concentrations of 30 and 800 ng/mL, respectively (Table 2). The mean matrix effects of ATBC were 42.1 ± 4.9% and 54.3 ± 1.2% at concentrations of 30 and 800 ng/mL, respectively.

#### 3.1.5. Stability

The stability of ATBC in plasma was investigated under various sample preparation and storage conditions. The resulting data are summarized in Table 3. In rat plasma samples, ATBC was stable for 8 h at room temperature (>94.1%, *n* = 5), and it was stable for 14 days at −20 °C (>99.9%, *n* = 5). It was also stable for at least three freeze-thaw cycles at −20 °C (>103.8%, *n* = 5). Furthermore, the prepared samples were stable when stored for 12 h at 4 °C in an autosampler (>103.2%, *n* = 5).

### 3.2. Pharmacokinetic Study

**Figure 3:**
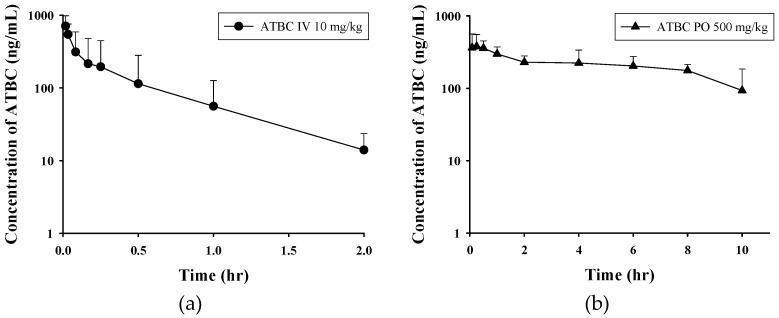
Plasma concentration-time profile of ATBC after (**a**) IV administration of 10 mg/kg of ATBC and (**b**) PO administration of 500 mg/kg to rats. Data were expressed as mean ± SD.

The developed method was successfully applied in a study of the pharmacokinetic parameters of ATBC in rat plasma after IV and oral administration. Plasma concentration-time curves are shown in Figure 3. The main pharmacokinetic parameters of ATBC are summarized in Table 3. After IV administration, ATBC was rapidly eliminated, with a clearance of 75.7 L/h/kg. As ATBC seems to follow two compartment model, initial (α) and final (β) excretion half-lives were calculated. To calculate initial and final phase half-lives, the first four points and the last three points were used respectively. When orally administered, ATBC was rapidly absorbed, with a Tmax of 0.4 h. The AUC of ATBC was 201.3 ng∙h/mL when administered IV (10 mg/kg), and it was 2757.1 ng∙h/mL when administered orally (500 mg/kg). Based on the dose normalized AUC, bioavailability was 27.4%. 

**Figure 4:**
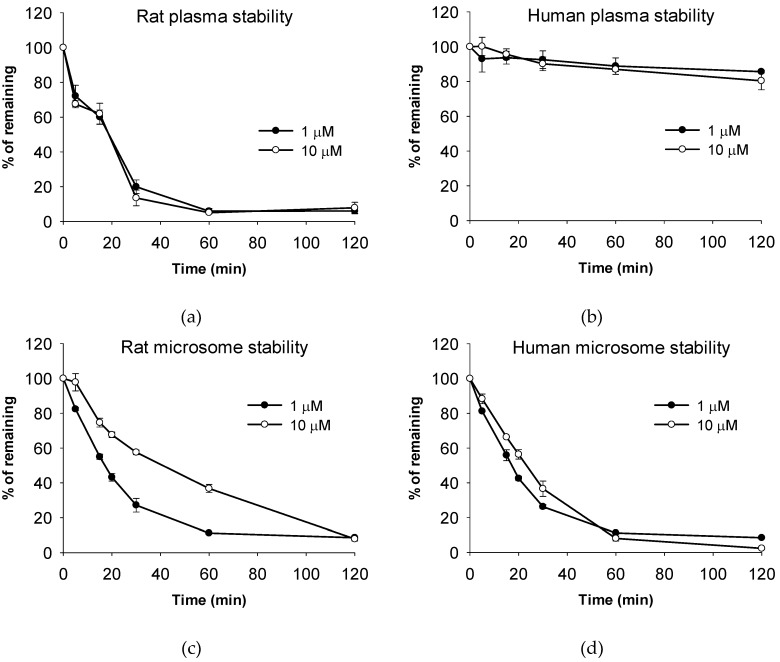
Metabolic stability of ATBC in plasma and liver microsomal fractions. (**a**) Rat plasma; (**b**) human plasma; (**c**) rat liver microsomes; (**d**) human liver microsomes. Data were expressed as mean ± SD (*n* = 3).

To better understand the pharmacokinetic behavior of ATBC, we tested the metabolic stability of ATBC in plasma and liver microsomes (Figure 4). In rat plasma, ATBC disappeared rapidly and remained less than 10% of the dose 1 h after incubation. The mean half-life was calculated to be 0.23 h and 0.21 h at 1 μM and 10 μM, respectively. This result was expectable and therefore we applied the esterase inhibitor in the sample preparation procedure. However, in human plasma, ATBC was fairly stable and the mean percentage of remaining was more than 80%. According to Bahar et al., there is species difference of plasma esterase expression and activity [16]. For example, paraoxonase and butyrylcholinesterase were highly expressed in humans and dogs, whereas carboxylesterase was only abundant in rabbits, mice, and rats. Thus, carboxylesterase is considered involved in hydrolysis of ATBC and accordingly, our results are well agreed with this. Therefore, such species difference should be considered for extrapolation of human pharmacokinetic parameters from the present data with rats. 

Meanwhile, the liver microsomal stability of ATBC was comparable between rats and humans; ATBC was easily metabolized in both microsomes. The mean half-life in rat liver microsomes was 0.27 h and 0.64 h at 1 μM and 10 μM, respectively. The mean half-life in human liver microsomes was 0.26 h and 0.37 h at 1 μM and 10 μM, respectively. Based on these findings, hepatic metabolism may play an important role in determining the systemic exposure of ATBC in humans.

**Figure 5:**
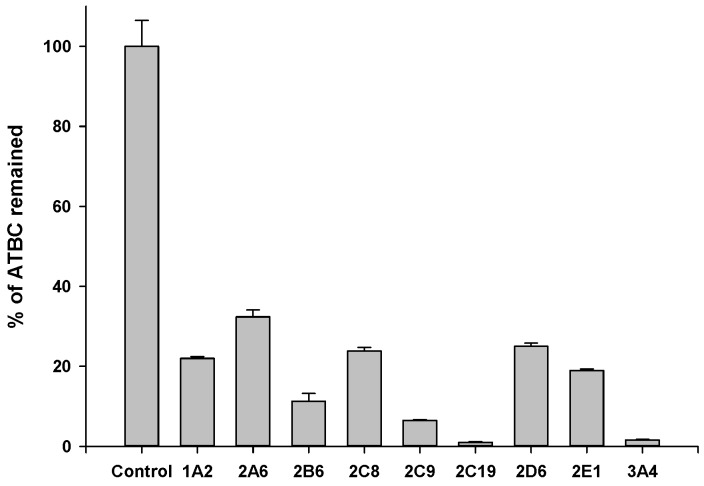
Metabolic stability of ATBC in human recombinant CYP supersomes. Data were expressed as mean ± SD (*n* = 3). ATBC was tested at 10 μM. Control: blank incubation matrix (all except for CYPs).

As described above, ATBC is fairly stable in human plasma, but easily metabolized in human liver microsomes. This indicates that liver microsomal enzymes may contribute substantially to ATBC metabolism in humans. To characterize the enzymes which may be involved in microsomal metabolism of ATBC, ATBC was incubated with human recombinant CYP supersomes and the stability was investigated. Figure 5 shows that all the CYP isozymes tested may be involved in the metabolism of ATBC. In particular, CYP 2C19 and CYP3A4 showed a high metabolic activity for ATBC.

## 4. Discussion

According to a report on the safety of ATBC, approximately 99% of 14C-ATBC orally administered to rats was excreted in urine (59–70%), feces (25–36%), and expired air (2%) within 48 h [3]. As reported in previous research, the metabolism of ATBC occurred rapidly in the present study. The metabolites mentioned in the previous report included acetyl citrate, monobutyl citrate, acetyl monobutyl citrate, dibutyl citrate, and acetyl dibutyl citrate [3]. Thus, the metabolites are mainly generated via hydrolysis and any significant toxic issues are not expected based on their chemical structures. However, inter-species differences in the pharmacokinetics and metabolism of ATBC need to be considered as the human profile may be different from the rat profile.

The IV plasma concentration patterns indicate that ATBC may follow the two compartment pharmacokinetic model. Thus, it suggests that ATBC is distributed to certain tissues at a slower rate. The pharmacokinetics of ATBC in oral administration was investigated at a somewhat higher dose (500 mg/kg) considering its detectability. In the plasma concentration profile of oral administration, the elimination appears to be nonlinear at some stages with individual variation in curve patterns. Thus, the elimination half-life for oral administration could not be calculated. This could be because the saturation in the processes of absorption, metabolism or elimination of ATBC takes place at a high dose. Therefore, the possibility of erroneous measurement of oral bioavailability should be considered. 

Rasmussen et al. investigated the effects of in vitro exposure to ATBC on ovarian antral follicle growth and viability [17]. At a dose of 0.01 μg/mL, ATBC increased the number of nongrowing follicles and terminal deoxynucleotidyl transferase dUTP nick end labeling (TUNEL)-positive area in treated follicles, pointing to the possibility that ATBC may disrupt antral follicle function at low concentrations. They also tested the effects of oral exposure to ATBC on female reproduction in mice [5]. Oral administration of ATBC at a dose of 10 mg/kg/day for 15 days decreased the number of primordial, primary and secondary follicles present in the ovary. These findings give rise to concern that low levels of ATBC may be harmful to ovarian function and affect female reproduction.

The findings in the aforementioned literature highlight the need to investigate possible toxic effects of ATBC at low dosages. A previous toxicological study found no adverse effects of ATBC (systemic toxicity or reproductive toxicity) at a relatively high dose (~1000 mg/kg/day) in animal models and concluded that ATBC was a safe alternative to phthalates. However, attention should still be paid to the safety of ATBC as it is extensively used in coating materials for tablets, capsules, and granules for masking the taste of medication or controlling drug release [18]. According to the FDA report, some patients may intake up to 60 mg of ATBC per day as a pharmaceutical excipient [2]. In this context, the pharmacokinetics of ATBC should be necessarily studied for its safe use. In addition, the exposure level of ATBC in plasma or other biofluids needs to be investigated.

## 5. Conclusions

We investigated the pharmacokinetic properties of ATBC in vitro and in vivo. ATBC was rapidly absorbed and eliminated and the bioavailability was 27.4%. Metabolic clearance may play an important role in determining the systemic exposure of ATBC. This is the first report to demonstrate the pharmacokinetic study of ATBC using LC-MS/MS. Our findings would be useful in studies investigating the safety and toxicity of ATBC.

## Figures and Tables

**Table 1:** Intra- and inter-day accuracy and precision for the determination of acetyl tributyl citrate (ATBC) in rat plasma.

Nominal Conc. (ng/mL)	Intra-Day (*n* = 5)			Inter-Day (*n* = 5)		
	Measured Conc. (ng/mL)	Accuracy (%)	Precision (%)	Measured Conc. (ng/mL)	Accuracy (%)	Precision (%)
10	11.8	117.6	6.8	10.3	103.4	11.4
30	31.2	103.8	5.4	31.2	104.1	5.0
400	405.1	101.3	4.9	411.7	102.9	4.8
800	725.9	90.7	11.4	801.3	100.2	6.2

**Table 2:** Stability of ATBC in rat plasma.

Stability	Recovery (% Remained)	
	Concentration (ng/mL)	
	30	800
Short-term (Room temperature for 8 h)	94.1 ± 7.6	94.9 ± 8.9
Long-term (−20 °C for 14 days)	100.6 ± 11.5	99.9 ± 14.5
Freeze-thaw (−20 °C, 3 cycles)	103.8 ± 10.7	104.2 ± 8.2
Auto-sampler (4 °C for 24 h)	106.8 ± 12.1	103.2 ± 10.8

**Table 3:** Pharmacokinetic parameters after intravenous (IV) and oral (PO) administration of ATBC to rats.

Parameter	ATBC	
	IV (10 mg/kg, *n* = 5)	PO (500 mg/kg, *n* = 6)
*t*_1/2α_ (h)	0.02 ± 0.01	-
*t*_1/2β_ (h)	0.58 ± 0.31	-
AUC (ng·h/mL)	201.3 ± 215.3	2757.1 ± 726.3
Tmax (h)	-	0.4 ± 0.3
Cmax (ng/mL)	-	478.7 ± 170.9
Vz (L/kg)	80.4 ± 47.4	-
Cl (L/h/kg)	75.7 ± 64.4	-
F (%)	-	27.4
